# Lignolytic Enzymes of a Mushroom *Stereum ostrea* Isolated from Wood Logs

**DOI:** 10.4061/2011/749518

**Published:** 2011-09-20

**Authors:** K. Praveen, B. Viswanath, K. Y. Usha, H. Pallavi, G. Venkata Subba Reddy, M. Naveen, B. Rajasekhar Reddy

**Affiliations:** ^1^Department of Microbiology, Sri Krishnadevaraya University, Anantapur 515055, Andhra Pradesh, India; ^2^Department of Environmental Science, Global College of Engineering and Technology, Kadapa 516162, Andhra Pradesh, India; ^3^Department of Botany, Sri Krishnadevaraya University, Anantapur 515055, Andhra Pradesh, India

## Abstract

Production of lignolytic enzymes by the mushroom fungus *Stereum ostrea* in liquid medium under conditions of vegetative growth was examined for 10 days in comparison to the reference culture *Phanerochaete chrysosporium*. Though growth and secretion of extracellular protein by *S. ostrea* were comparable to those of *P. chrysosporium*, yields of laccase enzyme by *S. ostrea* were higher than laccase titres of *P. chrysosporium* by more than 2 folds on the peak production time interval (IVth day of incubation). *S. ostrea* yielded titres of 25 units of laccase/ml as against 8.9 units of laccase/ml on the IVth day of incubation. *Stereum ostrea* also exhibited activities of other lignolytic enzymes, lignin peroxidase (LiP) and manganese peroxidase (MnP), higher than the reference culture. Growth of *S. ostrea* on the medium in the presence of Remazol orange 16 resulted in the decolourisation of dye, confirming the presence of lignolytic enzymes. *S. ostrea* appears to be a promising culture with complete lignolytic system.

## 1. Introduction

Lignin is the second most abundant aromatic polymer in nature with three-dimensional structure composed of phenyl propanoid units linked through several carbon-carbon and ether bonds [1, 2]. Such complex structure of lignin is designed in plant cell wall to protect plant cells from microbial attack [3]. Degradation of recalcitrant lignin requires an oxidative process mediated by lignolytic enzymes. Lignolytic enzymes include laccase (Lcc) (EC 1.10.3.2), lignin peroxidases (LiP) (EC 1.11.1.4), manganese peroxidases (MnP) (EC 1.11.1.3), and versatile peroxidases and are secreted by white rot fungi [2, 4, 5]. A few of them, in particular, *Phanerochaete chrysosporium* and *Trametes versicolor,* have been the focus of intensive research and a greater understanding of physiology biochemical and molecular biology of lignolytic enzymes in the above organisms have been gained [6]. Activities of lignolytic enzymes appear only in the culture medium after attainment of peak growth with exhaustion of nutrients—C, N, and S in respect of *P. chrysosporium* and *T. versicolor* [7]. Production of lignolytic enzymes in these organisms is enhanced by inducers [8]. Lignolytic enzymes in other organisms, *Cereporiopsis subvermispora*, *Trametes trogii* and *Panus tigrinus,* are constitutive and produced even under conditions of nitrogen sufficiency [9–11]. Profiles of enzymes of lignolytic system depend on growth conditions and vary from one organism to another. Peroxidases are dominant in lignolytic system in respect of *P. chrysosporium,* where laccase is a major component in lignolytic system of *Ganoderma adspersum* [6, 12, 13]. In view of broader specificity and oxidation of wider range of xenobiotic compounds including chlorinated phenolics, synthetic dyes, pesticides, and polycyclic aromatic hydrocarbons, lignolytic enzymes offer advantages for biotechnological applications. Although the majority of earlier studies have been on lignin-degrading enzymes of organisms, *P. chrysosporium*, *Pleurotus ostreatus* and *Trametes versicolor*, there has been a growing interest in studying lignolytic enzymes of wider array of white-rot fungi from the standpoint of comparative biology but also with expectation of finding better lignin degrading system. Activity of laccase enzyme was detected in the culture filtrate of *Stereum ostrea* [14]. In that direction, the present investigation has been undertaken to study lignolytic enzymes of a mushroom, *Stereum ostrea* in comparison to the reference culture *Phanerochaete chrysosporium*. 

## 2. Materials and Methods

*Stereum ostrea* was kindly supplied by Professor M. A. Singaracharya, Department of Microbiology, Kakatiya University, Andhra Pradesh, India, and was isolated from wood logs. The reference culture, *Phanerochaete chrysosporium* was obtained from IMTECH, India. Both the cultures were maintained on Koroljova-Skorobogat'ko medium [15] because of good growth [14].

Sterile Koroljova-Skorobogat'ko medium was dispersed into sterile 250 mL Erlenmeyer flasks at a rate of 50 mL of medium per flask. The flasks were inoculated with homogenized mycelial suspension and incubated in an orbital shaker (Orbitek, Chennai, India) at 30°C and speed of 200 rpm. The flasks with growing cultures of *Stereum ostrea* and *Phanerochaete chrysosporium* were withdrawn at different time intervals during the course of the experiment for processing. The entire culture medium in flasks was used for processing in the same manner as mentioned earlier [14]. The fungal cultures were aseptically filtered through preweighed Whatman no 1 filter paper to separate mycelial mat and the culture filtrate. The filter paper along with mycelial mat was dried at 70°C in an oven until constant weight. Difference between the weight of the filter paper having mycelial mat and weight of only filter paper represented biomass of fungal mat. Fungal growth was expressed in terms of mg/flask. pH of the culture filtrate was measured. Content of extracellular protein in culture filtrates of both fungi was estimated according to Lowry et al. [16]. 

### 2.1. Enzyme Assay

Activities of lignolytic enzymes in the cultural filtrate of both fungal cultures were estimated following the standard protocols. Laccase activity was assayed using 10 mM guaiacol in 100 mM acetate buffer (pH 5.0) containing 10% (V/V) acetone. The change in absorbance of the reaction mixture containing guaiacol was monitored at 470 nm (*ε* = 6740 M^−1^ cm^−1^) for five minutes of incubation [17] Laccase activity was expressed in International Units (IU), where one unit corresponded to the amount of enzyme that oxidized one micromole of guaiacol per minute. Lignin peroxidase activity was determined by oxidation of veratryl alcohol at 310 nm (*ε* = 9,300 M^−1^ cm^−1^) [18]. The reaction mixture was composed of 0.5 mL culture filtrate, 0.4 mM H_2_O_2_ and 50 mM tartaric acid (pH 2.5) and 2 mM veratryl alcohol. The enzyme activity was expressed in IU, where one unit of LiP corresponded to the amount of enzyme that oxidized one micromole of veratryl alcohol per min. MnP activity was determined by oxidation of phenol red at 610 nm [19]. The assay mixture included 0.5 mL culture filtrate, 0.25 M sodium lactate (pH 4.5), 0.5% bovine albumin, 200 mM MnSO_4_, 2.0 mM H_2_O_2_ (prepared in 0.2 mM sodium succinate buffer pH 4.5) and 0.1% phenol red. The changes in the absorbance of reaction mixture was monitored at 610 nm (*ε* = 22,000 M^−1^ cm^−1^) for 5 min. MnP activity was expressed in IU, where one unit of MnP was defined as the amount of enzyme that oxidized one micromole of phenol red per min.

### 2.2. Decolourisation of Dye

Another experiment was conducted by growing both fungal cultures in the same liquid medium in the presence of dye Remazol orange 16 (*λ*max  = 530 nm) at concentration within a range of 0.02 to 0.10% in 250 mL Erlenmeyer flasks in the same manner as mentioned earlier. Medium without dye and inoculum and dye-amended medium without inoculum were maintained as controls. At regular intervals flasks were withdrawn for processing for determination of decolourisation of dye in addition to parameters mentioned in the previous experiment. Absorbance of colour of dye in the uninoculated medium amended with dye was measured against uninoculated medium without dye at 530 nm at any given time interval and is treated as absorbance of control. Absorbance of colour of dye in the culture filtrate derived from the growth of fungi was measured against uninoculated medium without dye at 530 nm at the respective time interval and was considered as observed absorbance. Decolourisation was expressed as activity (%)

(1) $$\begin{array}{cc} & \text{\%}\;\text{Decolourisation}\\ & \;=\frac{\text{Control}\;\text{absorbance}-\text{observed}\;\text{absorbance}}{\text{Control}\;\text{absorbance}}\times 100 \end{array}$$

## 3. Results and Discussion

**Figure 1:**
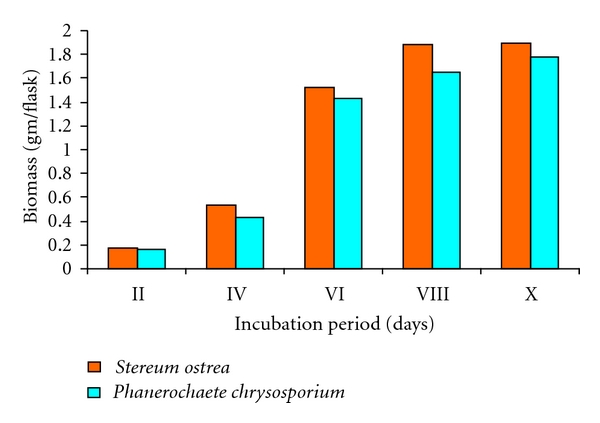
Biomass of the fungal cultures.

Biomass of cultures of *Stereum ostrea* and *Phanerochaete chrysosporium* upon growth in liquid medium under shaking conditions was determined and is presented in (Figure 1). Growth of both cultures was initially slow for 4 days and then picked up and remained steady from 8th day of incubation. *Stereum ostrea* produced maximum biomass of 1.89 g/flask on the 10th day of incubation as against 1.78 g/flask in respect of *P. chrysosporium*. 

**Figure 2:**
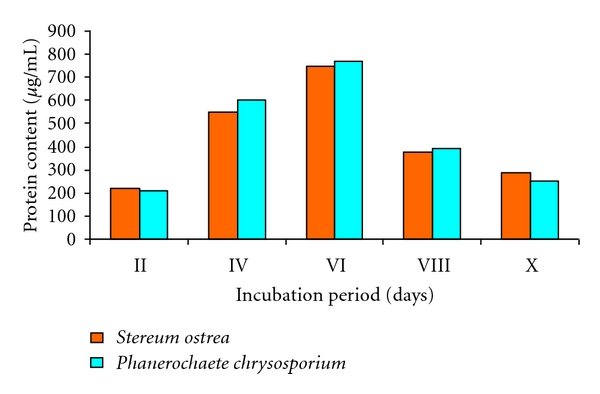
Extracellular protein of the fungal cultures.

The secretion of extracellular protein into liquid medium under shaking conditions for 10 days was measured (Figure 2). The secretion of extracellular protein by both fungal cultures increased with increase in incubation time and reached maximum on 6th day of incubation and thereonwards dropped. *Stereum ostrea* secreted maximum protein content of 750 *μ*g/mL into medium as against 770 *μ*g/mL by *P. chrysosporium* on 6th day of incubation.

Wood-rot fungi are a large group of microorganisms with a potential to metabolise lignin by action of three major groups of enzymes: Lignin peroxidase, Mn peroxidase, and Laccase outside cell. Our knowledge in the understanding of nutritional requirements for growth of the organisms, *Phanerochaete chrysosporium*, and *Trametes versicolor*, *Pleurotus ostrea*, *Trametes trogii*, has been improving with continuous efforts of probing. There are many wood-rot organisms which have not been explored. The present study examined the growth of an unexplored wood-rot fungus-*Stereum ostrea,* in comparison to the model lignolytic culture *P. chrysosporium*. Both the test organism and the model culture grew well on Korlojova liquid medium used in this study under shaking and noninducing conditions as reflected by large biomass of both cultures and high protein secretion.

**Figure 3:**
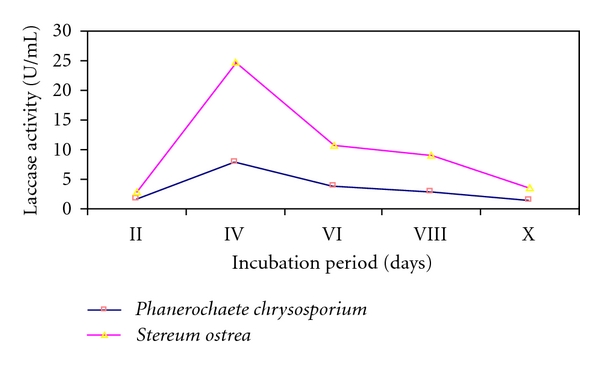
Laccase activity of the fungal cultures.

Both cultures exhibited laccase activity when grown on medium under noninducing conditions (Figure 3). Unlike extracellular protein secretion, laccase production by both cultures touched peak on 4th day of incubation and thereonwards declined. *Stereum ostrea* gave titres of laccase 3 times higher than *P. chrysosporium*. Maximum yields of laccase to the tune of 25 Units/mL by *S.ostrea* was recorded as against only 9.0 units/mL by *P. chrysosporium*. Thus, results clearly show that *S. ostrea* was better than the reference culture on the score of laccase production.

Production of lignolytic enzymes was studied in only a few wood-rot organisms: *Phanerochaete chrysosporium*, *Trametes versicolor*, *Pleurotus ostrea, *and *Trametes trogii*. *Stereum ostrea* notably displayed higher capacity of laccase production (25 Units/ml) under noninducing conditions than even other white-rot fungi *Ganoderma sp*. [13], newly isolated basidiomycete PM1 [20] and *Trametes versicolor* [21] and *Trametes hirsuta* [22] reported elsewhere. Baldrain and Šnajdr [23] compared the production of laccase by litter-decomposing basidiomycetes with reference white-rot fungi *Trametes versicolor* and *Pleurotus ostreatus* on HNHC medium and found yields of laccase by only one basidiomycete *Collybia dryophila* close to figures of laccase yields of *Trametes versicolor* (60 Units/lit). Growth of the white-rot fungus *Coriolopsis rigida* [24] and *Trametes trogii* [10] in liquid medium under induced conditions produced maximum levels of 40 Units/ml and 90 Units/ml of laccase activity, respectively. In the majority of the above studies, laccase assay was determined with use of 2,2′-Azino-bis(3-Ethylbenzthiazoline-6-Sulfonic Acid) as a substrate, whereas guaiacol was employed as substrate in assay medium for laccase in the present study. As Kcat of laccase of different organisms *Pleurotus ostreatus* POXA and *Trametes trogii* with substrate ABTS was higher than Kcat of laccase of the same organisms with guaiacol as substrate, yields of laccase in cultures of organisms determined on the basis of ABTS method is expected to be higher [25]. This fact is taken into consideration along with production of laccase carried out under noninduced conditions in the present study, yields of laccase by *Stereum ostrea *were comparable and may be even higher than yields of laccase by *C. rigida* and *Trametes trogii*. 

**Figure 4:**
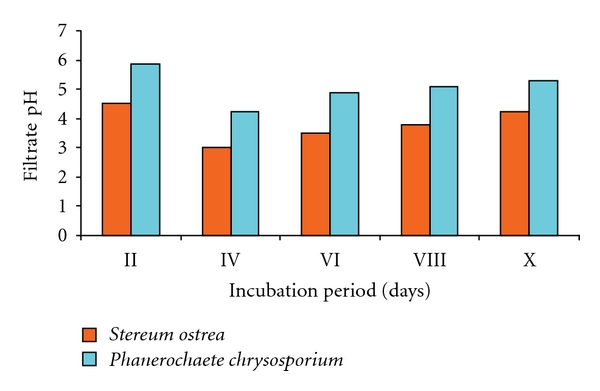
pH of the fungal cultures.

Growth of both fungal cultures resulted in drop in pH of Koroljova medium which was initially set to pH 6.0 (Figure 4). During the course of growth of fungal cultures, pH of culture medium was not regulated. Decrease in pH of the culture medium of both fungal cultures occurred up to 4th day of incubation, and there was recovery in pH of the culture medium towards the end of the experiment. Drop in pH of the medium was sharper in respect of *S. ostrea* and fell below 4 on the 4th day of incubation.

Another experiment was conducted with the selected fungal cultures in liquid Koroljova medium to find out whether other lignolytic enzymes lignin peroxidase and manganese peroxidase are present in lignolytic system of *S. ostrea*. LiP activity was detected in the culture filtrate of *S. ostrea* and *P. chrysosporium* throughout the incubation period (Table 1). There was an increase in activity of LiP up to 6th day of incubation followed by declining trend. Maximum activities of LiP recorded in respect of *S. ostrea* and *P. chrysosporium* on 6th day of incubation were 0.516 and 0.472 U/mL, respectively. *Stereum ostrea* displayed activity of even LiP on higher side than *P. chrysosporium.*

Like LiP, Mn peroxidase of both cultures followed the similar trend during the course of incubation (Table 2). Both cultures secreted MnP into broth throughout the incubation period. But maximum activities of MnP in both cultures was observed on 6th day of incubation. *S. ostrea* exhibited MnP activity two folds higher than *P. chrysosporium*. *S. ostrea* yielded titres of 0.590 U/ml of MnP as against titres of 0.272 U/mL by *P. chrysosporium* on 6th day of incubation. 

Breakdown of lignin is mediated by action of the enzymes lignin peroxidase, and manganese peroxidase apart from laccase. Titres of both LiP and MnP yielded by both cultures under noninducing conditions were low when compared to laccase by the same cultures and did not exceed one Unit/ml. However, these yields of lignolytic peroxidases by *S. ostrea* in the present study were considerable in comparison to other organisms including *P. chrysosporium* on different growth media under different conditions. Growth of *P. chrysosporium* in submerged fermentation generated MnP and LiP with specific activity of 144 and 14 U/mg, respectively [26]. Yields of lignolytic peroxidase enzymes in majority of studies with different strains of *P. chrysosporium* in liquid medium occurred within a range of 0.07–0.8 U/mL [12, 27–30]. Growth of other organisms—*Trametes versicolor* [23, 31], hyperlignolytic fungus IZU-154 [32], the strain K_1_ isolated from polyphenol polluted site [33], *Nematoloma forwardii* [34], and *Pleurotus pulmonarius* [35] in solid state fermentation/submerged fermentation produced lignolytic peroxidases at low levels. However, two organisms *Phellunus robusties* [13] and *Schizophyllum commune* [27], with high production of MnP in liquid medium to the extent of 10 and 580 U/mL, respectively were spotted in the literature. Differences in titres of enzyme yielded by organisms in different studies may be due to differential inherent capacity of organisms to synthesize lignolytic enzymes, growth conditions, nutritional requirements and inducer. Organism like *P. chrysosporium* produced higher yields of lignolytic enzymes under conditions of starvation for nitrogen and carbon [36, 37], whereas in other cases—*Panus tigrinus* lignolytic enzymes were generated under even conditions of nitrogen sufficiency. The presence of inducers veratryl alcohol induced 2-fold increase in yields of lignolytic enzymes by *P. chrysosporium *[37]. Yields of lignolytic enzymes by *S. ostrea* in the present study were determined only under noninducing conditions. Exposure of *S. ostrea* to inducer may further improve yields of lignolytic enzymes. Laccase appears to be a dominant component in lignolytic enzymes of *S. ostrea* under growth conditions employed in the present study. Similar observation of dominance of laccase in lignolytic system of *Ganoderma adspersm* was made [13]. In contrast, lignolytic peroxidases are major component of lignolytic system of *P. chrysosporium Schizophyllum commune* [27].

Textile dye Remazol orange-16, has undergone decolorisation even at the highest concentration (0.10%) in both grown cultures (Table 3). Decolourisation of dye by both cultures followed the pattern of growth. Decolourisation was initially slow later picked up and reached maximum on VI day of incubation in both cultures but values were lower side in case of *Phanerochaete chrysosporium*.

Maximum decolorisation of dye by *Stereum ostrea *at 3 different concentrations—0.01, 0.05, and 0.10% was found to be 84.42, 81.27, and 70.85%, respectively, where as the corresponding figures in respect of *Phanerochaete chrysosporium* was 77.66, 66.74, and 65.47 at the 6th day of incubation.

Decolourisation of dye Remazol orange-16 in the present study by both cultures indicates indirect evidence for presence of lignolytic enzymes in the culture filtrates of both cultures used in the present study. Activities of lignolytic enzymes Lcc, MnP, and LiP in the culture filtrate of both fungal cultures grown on the medium in the presence of Remazol orange-16 at regular intervals were measured. As activities of these enzymes in the culture filtrate, derived from growth of fungi in the medium amended with dye, followed the similar trend to those of the same enzymes in the culture filtrate of the same cultures grown in the medium without dye, the results are not represented here. Colour changes of dyes may also occur due to sensitivity of dyes to pH changes that took place in medium upon growth of fungal cultures. It was tested whether Remazol orange-16 undergoes change in colour in a medium with pH up to 3. The possibility of decolourisation due to pH changes was ruled out because of stability of colour of Remazol orange-16 under low pH. Generally, decolorisation of dyes is probably due to physical adsorption of dye to mycelial mat or participation of lignolytic enzymes or combination of both. Appearance of colour on mycelial mat followed by loss of colour from mycelial mat was an observation made in the present study and supports involvement of lignolytic enzyme in decolorisation of dye. Similarly, a clearance of purple colour around fungal growth on agar medium with poly-R Assay was considered as a positive result for production of lignolytic enzymes and was used for screening basidiomycetes for the presence of lignolytic enzymes [35]. Decolourisation of dyes was also demonstrated even with purified lignolytic enzyme Laccase [14]. For this simple reason, protocols with use of dyes as possible substrate for lignolytic enzymes have been developed and permit rapid assay of lignolytic enzymes [8, 38].

## 4. Conclusions

The following conclusions can be drawn from the results of the present study. The white-rot fungus *S. ostrea* produces a complete lignolytic system Lcc, LiP, and MnP under conditions of vegetative growth. Lcc appears to be a dominant component in the lignolytic system of *S. ostrea*. For production of lignolytic enzymes, *Stereum ostrea* culture is more promising and potential culture than the reference culture *P. chrysosporium.*

## Figures and Tables

**Table 1:** Lignin peroxidase activity of the fungal cultures.

Incubation Period (days)	Lignin peroxidase activity (U/mL) of	
	*S. ostrea*	*P. chrysosporium*
II	0.300	0.216
IV	0.432	0.344
VI	0.516	0.472
VIII	0.260	0.241
X	0.172	0.165

**Table 2:** Manganese peroxidase activity of the fungal cultures.

Incubation Period (days)	Manganese peroxidase activity (U/mL) of	
	*S. ostrea*	*P. chrysosporium*
II	0.164	0.036
IV	0.292	0.220
VI	0.590	0.272
VIII	0.328	0.200
X	0.200	0.144

**Table 3:** Decolorisation of textile dye, Remazol orange, 16 by the fungal cultures.

Incubation period (days)	% decolorisation of dye by					
	*Stereum ostrea* at			*Phanerochaete chrysosporium* at		
	0.01%	0.05%	0.10%	0.01%	0.05%	0.10%
II	31.12	29.15	28.42	17.52	15.25	10.76
IV	77.31	70.56	62.80	70.56	68.13	60.75
VI	84.42	81.27	70.85	77.66	66.74	65.47
VIII	81.17	75.26	60.13	71.19	60.11	58.75
X	81.18	75.63	60.97	71.31	60.20	58.85
